# Clinical relevance and outcome of routine endomyocardial biopsy to detect rejection after heart transplantation

**DOI:** 10.1016/j.jhlto.2025.100320

**Published:** 2025-06-14

**Authors:** Leendert C. Kieviet, Steven A. Muller, Mariusz K. Szymanski, Manon G. van der Meer, M. Louis Handoko, Saskia Z.H. Rittersma, Saskia C.A. de Jager, Egidius E. van Aarnhem, Annelotte Vos, Pim van der Harst, Linda W. van Laake, Marish I.F.J. Oerlemans

**Affiliations:** a Department of Cardiology, University Medical Center Utrecht, Utrecht, The Netherlands; b Division of Cardiology, Department of Medicine, Johns Hopkins University School of Medicine, Baltimore, MD; c Transplantation Center University Medical Center Utrecht, Utrecht, The Netherlands; d Laboratory of Experimental Cardiology, University Medical Center Utrecht, Utrecht University, Utrecht, The Netherlands; e Circulatory Health Research Center, University Medical Center Utrecht, Utrecht University, Utrecht, The Netherlands; f Department of Cardio-Thoracic Surgery, University Medical Center Utrecht, Utrecht, The Netherlands; g Department of Pathology, University Medical Center Utrecht, Utrecht, The Netherlands

**Keywords:** Heart transplantation, Endomyocardial biopsy, Acute cellular rejection, Complications, Biomarkers

## Abstract

**Background:**

Endomyocardial biopsy has been the cornerstone of monitoring rejection after heart transplantation for decades. Although recommendations advise routine biopsies during the first 3-12 months, this timeframe is broad, and intercenter variability persists in its application. Here, we report the yield and complication rate of routine endomyocardial biopsies during the past 36 years of post-transplantation care to monitor acute cellular rejection.

**Methods:**

In this retrospective, single-center study, we collected all routine biopsy data after transplantation between 1986 and 2022. The total number of biopsies, type of rejection, complications, and survival were analyzed in the total population as well as per different endomyocardial biopsy protocol over time period (Period 1: 1986-1994; Period 2: 1994-2009; Period 3: 2009-2022).

**Results:**

In 474 patients (71.1% male, age at transplant 47.7 ± 12.6 years), 8185 routine biopsy procedures were performed: 29.9 ± 11.1 per patient for Period 1 (*n* = 83), 16.9 ± 3.8 for Period 2 (*n* = 220) and 11.6 ± 2.4 for Period 3 (*n* = 171). Complication rate was low (1.7%; *n* = 139/8185) and 19.8% (*n* = 94/474) patients experienced clinically-relevant rejection (≥2R) which mainly occurred <6 months post-transplantation (89.4%; *n* = 84/94). The incidence of rejection decreased over time, leading to an improved rejection-free survival (*p* < 0.001) with a subsequent increase in Number-Needed-to-Diagnose. Importantly, severe acute cellular rejection did not occur in Period 3 in the first year post-transplantation.

**Conclusion:**

Acute cellular rejection, including clinically-relevant rejection, has declined significantly over time and is rare beyond 6 months post-transplantation. A low-frequency approach seems feasible and safe, which is relevant for the transition towards less-invasive protocols to detect rejection, especially early post-transplantation.

## Graphical abstract

**Figure:**
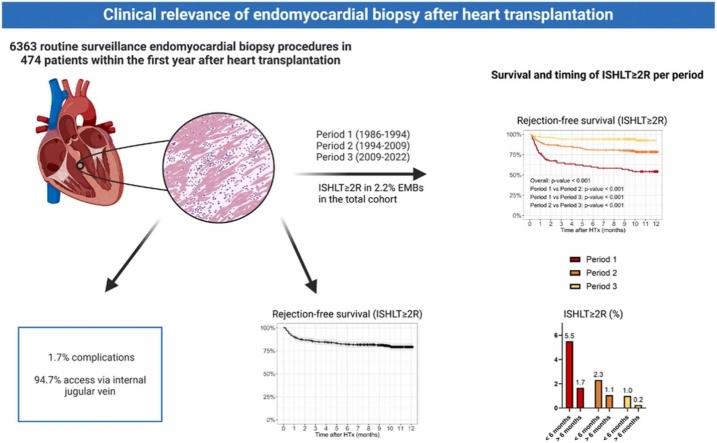

## Background

Early detection of acute cellular rejection (ACR) in patients after heart transplantation (HTx) occurs in up to 13% of patients within the first year and is important to preserve graft function and improve long-term survival.^1^ Endomyocardial biopsy (EMB) has been the cornerstone of monitoring rejection for decades and provides a detailed histopathologic diagnosis with well-established prognostic and therapeutic implications.^2^ Nevertheless, a clear consensus on the optimal frequency and timing of EMB is lacking, leading to various center-specific protocols, with some centers performing EMBs for even years post-HTx.^3^, ^4^ Being an invasive procedure, EMB is associated with serious complications such as cardiac tamponade, hematoma, and tricuspid valve regurgitation.^5^ Furthermore, patient discomfort and psychological impact, the low sensitivity of detecting antibody-mediated rejection, and the variation in interpretation by cardiovascular pathologists are limitations of invasive rejection monitoring by EMB.^6^, ^7^

Non-invasive biomarkers and techniques (ie, gene expression profiling, cell-free DNA, novel imaging techniques) have now become available to detect allograft rejection and are increasingly utilized in the USA.^8^, ^9^ However, the practical implementation is hindered by significant limitations, including its use in the early period after HTx, costs, necessary infrastructure, and differences in the diagnostic threshold for ACR and antibody-mediated rejection post-HTx.^10^, ^11^, ^12^ As such, in most European HTx-centers, EMB remains the gold standard for surveillance and clinical decision-making, showing a distinct difference in rejection management between the USA and Europe.

With the increasing demand on health care resources in general, there is a clear need for data to determine the optimal surveillance frequency considering both the risk of ACR detected by EMB and the feasibility to transition towards less-invasive surveillance protocols. The purpose of this study is to describe (1) the diagnostic yield of EMB, (2) the complication rate of EMB, and (3) to contribute to the determination of the optimal surveillance frequency for rejection detection. Here, we report our single-center experience of ACR detection using routine surveillance EMBs during the past 36 years in a large population of heart transplant recipients.

## Methods

### Study population and data collection

The study population was recruited from the University Medical Center Utrecht, The Netherlands. We identified all HTx patients between 1986 and 2022 who both underwent HTx and attended follow-up at our center.^13^ The medical history of each patient was obtained by review of medical records, clinical evaluation, and patient interview. Detailed clinical information regarding demographics, rejection, and complications of all EMBs were collected, as described below. This study followed the Code of Conduct and the Use of Data in Health Research and was approved by local ethics and/or institutional review boards (UCC-UNRAVEL #12-387). Furthermore, all heart transplant recipients in the Netherlands provided written informed consent for collection of clinical data as part of a national ongoing quality improvement program as previously described.^14^

### Routine EMB protocols

In the past 36 years of HTx care at the UMC Utrecht, three periods were distinguished based on the number of routine biopsy procedures performed (excluding symptom-triggered biopsies), an overview is provided in Supplementary Table 1. Briefly, the main difference between the periods is that over time, the number of routine EMB procedures decreased with improving clinical experience in post-transplant care. Furthermore, the use of routine surveillance EMB > 1 year post-HTx became less frequent and was abandoned in the most recent period (Period 3).

### Classification of ACR

ACR was classified by an experienced pathologist panel according to the current International Society of Heart and Lung Transplantation (ISHLT) guidelines.^15^ In short, ACR was classified in a) grade 1R, defined as interstitial and/or perivascular infiltrate with up to one focus of myocyte damage (ie, mild ACR); b) Grade 2R defined as ≥2 foci of infiltrates with associated myocyte damage (ie, moderate ACR); and c) Grade 3R, defined as diffuse infiltrate with multifocal myocyte damage, with or without edema, hemorrhage, or vasculitis (ie, severe ACR). For the purpose of this study, we combined moderate and severe ACR into “clinically-relevant” ACR (ie, ISHLT ≥2R), as in general only moderate and severe ACR warrants medical intervention in daily clinical practice. Lastly, we stratified the occurrence of ACR into early and late rejection, defined as <6 months and between 6 and 12 months after HTx, respectively.

### Classification of complications

Complications were defined as a composite of death, pericardial tamponade, hemothorax, pneumothorax, tricuspid valve injury, access-site hematoma, (supra)ventricular arrhythmias, and cardiac embolism. Subsequently, complications were classified based on their severity into major (a composite of death, pericardial tamponade, intrathoracic vascular injury, and pneumothorax) or minor (a composite of access-site hematoma, [supra]ventricular arrhythmias, tricuspid valve injury, or embolism) complications.

### Study outcomes

The primary endpoints of this study were early (<6 months post-HTx) and late rejection (>6 months post-HTx) for both any ACR and clinically-relevant ACR (ie, moderate and severe rejection, ISHLT ≥ 2R). The secondary endpoints of this study were major and minor complication.

### Statistical analysis

Nominal variables were expressed as number (%), and continuous variables as mean ± standard deviation or median (interquartile range), as appropriate. Comparisons for binary variables were performed by Chi-square or Fisher’s exact test. For continuous variables, independent *t*-test or Mann-Whitney *U* test were used. Kaplan-Meier curves were utilized to assess the within-patient survival free probability from the primary endpoints. Differences between periods were calculated using the log-rank test. To determine the risk of ACR and complications from performing routine EMBs, we calculated incidence rates for both. A *P* < 0.05 was considered statistically significant throughout. All data were analyzed using R Statistical Software (v4.3.3; R Core Team 2024.).

## Results

### Study population

**Figure 1:**
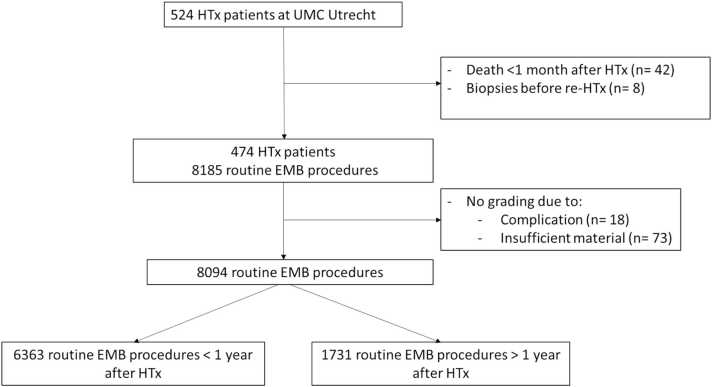
*Flowchart patient and EMB inclusion.* HTx, heart transplantation; EMB, endomyocardial biopsy.

As shown in Figure 1, 524 patients underwent a HTx with subsequent follow-up at the UMC Utrecht between 1986 and 2022. In total, 50 patients (9.5%) were excluded due to non-rejection related death or re-HTx within 6 months, resulting in a total of 474 patients (mean age 47±12 years; 28.19% female) with follow-up routine EMB data in the first year post-HTx. The most common etiology for HTx was dilated cardiomyopathy, followed by ischemic cardiomyopathy (Table 1). As expected, ischemic cardiomyopathy was replaced by dilated cardiomyopathy as the most common etiology over time.

**Table 1:** Patient Characteristics

Patient characteristics	Total (*n* = 474)	Period 1: 1986-1994 (*n* = 83)	Period 2: 1994-2009 (*n* = 220)	Period 3: 2009-2022 (*n* = 171)
*Age at transplant (SD)*	47.7 (12.6)	47.0 (9.1)	47.1 (13.3)	48.9 (13.2)
*Sex (% female)*	137 (28.9)	19 (22.9)	59 (26.8)	59 (34.5)
*Primary cardiac diagnosis*				
Dilated cardiomyopathy	212 (44.7)	18 (21.7)	92 (41.8)	102 (59.6)
Ischemic cardiomyopathy	190 (40.1)	46 (55.4)	103 (46.8)	41 (24.0)
Hypertrophic cardiomyopathy	24 (5.1)	3 (3.6)	11 (5)	10 (5.8)
Other	48 (10.1)	16 (19.3)	14 (6.4)	18 (10.5)

### EMB procedures

A total of 8185 EMB procedures were classified as routine surveillance biopsy procedures, with an average of 4-5 biopsies of the right ventricular endomyocardium per procedure. The average number of routine EMB procedures per patient were 29.9 ± 11.1 for Period 1 (*n* = 83, 1986-1994), 16.9 ± 3.8 for Period 2 (*n* = 220, 1994-2009), and 11.6 ± 2.4 for Period 3 (*n* = 171, 2009-2022), as shown in Supplementary Table 1.

1.1% of all biopsy procedures did not lead to biopsy grading due to procedural complications (*n* = 18) or because of insufficient biopsy material (*n* = 73; Figure 1). The remaining EMB procedures were analyzed for the occurrence of ACR based on the judgment of the local pathologist. Since the protocols involved varying numbers of EMBs beyond the first year post-HTx, further analysis was limited to EMBs collected within the first 12 months after HTx. Of note, the incidence of clinically-relevant rejection (ISHLT ≥ 2R) in routine surveillance EMB procedures >12 months after HTx (standard clinical practice in Period 1; *n* = 1731) was low (1.1%), given the incidence of 4.4% with the first 12 months in the same period (Supplementary Table 2).

### ACR in the first year after HTx

**Figure 2:**
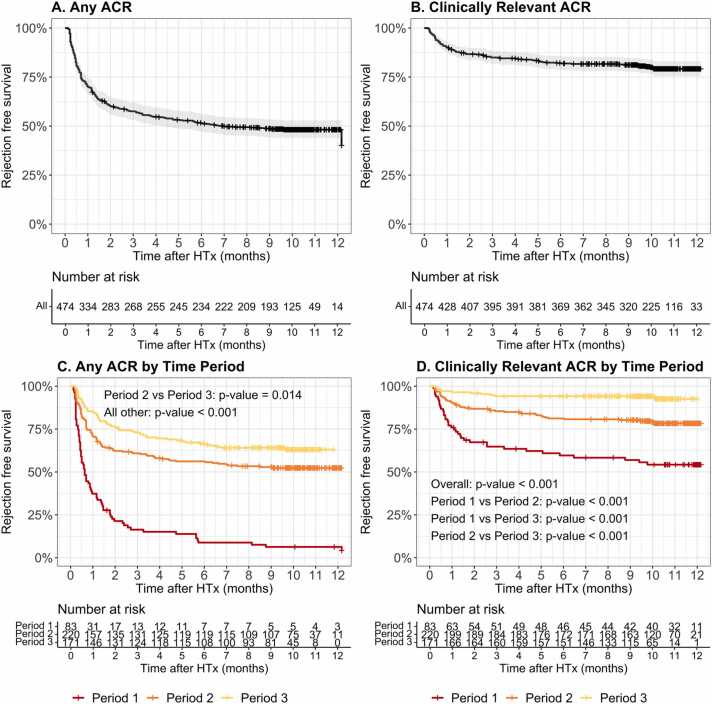
*Rejection-free survival post-HTx.* ACR-free survival post-HTx for **(A)** any ACR; **(B)** Clinically-relevant ACR; **(C)** any ACR by time period; and **(D)** clinically-relevant ACR by time period. Red, orange, and yellow lines depict Period 1, Period 2, and Period 3, respectively. HTx, heart transplantation.

In all EMB procedures performed, any ACR (ISHLT ≥ 1R) was detected in 11.1% of cases, while clinically-relevant (ISHLT ≥ 2R) ACR occurred in 2.2% (Supplementary Table 2). As shown in Supplementary Table 2, clinically-relevant ACR was detected in 4.4% of EMBs in Period 1, 2.0% in Period 2% and 0.8% in Period 3. When considering individual patients, any ACR (ISHLT ≥ 1R) occurred in 51.5% of cases (*n* = 244/474) and clinically-relevant ACR (ISHLT ≥ 2R) occurred in 19.8% (*n* = 94/474) of cases across our entire cohort. The rejection-free survival at 12 months for any ACR and clinically-relevant ACR was 48.5% (*n* = 230/474) and 80.2% (*n* = 380/474), respectively (Figure 2A and B). Both any ACR and clinically-relevant ACR showed a significant decrease over time (Figure 2C and D; *p* < 0.001).

### Early vs late ACR

**Figure 3:**
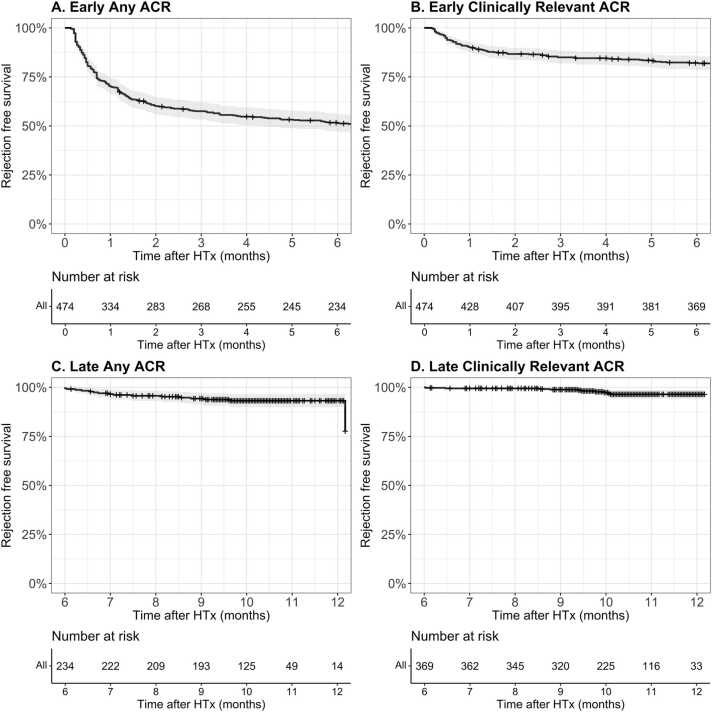
*Early and late rejection-free survival post-HTx.* ACR-free survival post-HTx for **(A)** early any ACR; **(B)** early clinically-relevant ACR; **(C)** late any ACR; and **(D)** late clinically-relevant ACR. HTx, heart transplantation.

To assess in which timeframe post-HTx (early vs late) the largest decrease in rejection occurred, we analyzed early vs late ACR per period. Within the first 6 months post-HTx, any ACR was observed in 48.1% (*n* = 228/474) of the patients, and clinically-relevant ACR in 17.7% (*n* = 84/474) of the patients (Figure 3A and B). Consequently, the majority of ACR episodes occurred within the first 6 months post-HTx, with 93.4% (*n* = 228/244) of any ACR and 89.4% (*n* = 84/94) of clinically-relevant ACR occurring in this early period. In contrast, late ACR (>6 months post-HTx) was less prevalent, with any ACR occurring in only 6.8% of cases (*n* = 16/234) and clinically-relevant ACR in 2.7% of cases (*n* = 10/369) (Figure 3C and D).

**Figure 4:**
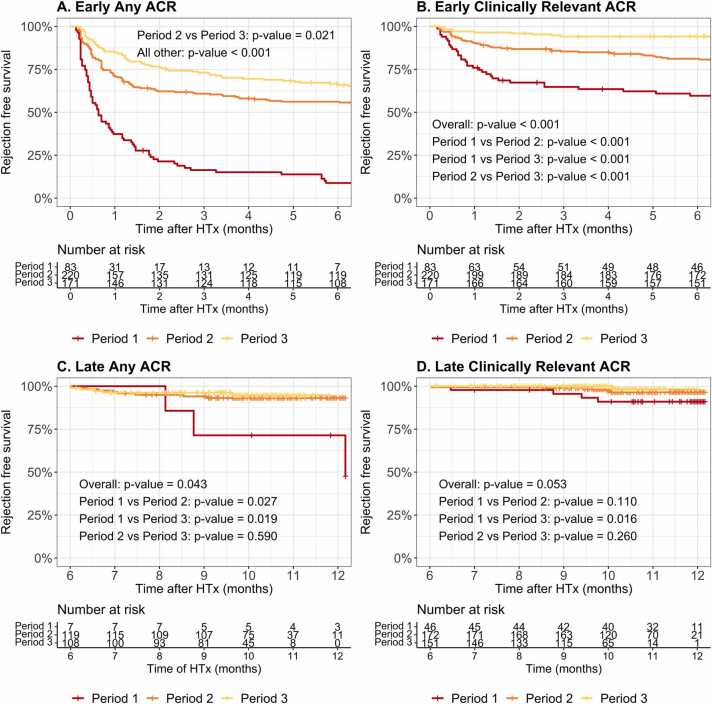
*Rejection-free survival early (<6 months) and late (>6 months) post-HTx per Period.* ACR free survival post-HTx for **(A)** early any ACR by time period; **(B)** early clinically-relevant ACR by time period; **(C)** late any ACR by time period; and **(D)** late clinically-relevant ACT per Period. Red, orange, and yellow lines depict Period 1, Period 2, and Period 3, respectively. HTx, Heart transplantation.

To better understand the decrease ACR episodes within the first year post-HTx over time, we further analyzed the differences per period for both early and late ACR. Both any ACR and clinically-relevant ACR decreased significantly over time post-HTx, mainly driven by a substantial reduction in early ACR, while late ACR (>6 months) remained relatively stable over time (Figure 4). Importantly, severe ACR (ISHLT 3R) was absent in the most recent period (Supplementary Table 3**;** Period 3, 2009-2022).

### Complications

Two different vascular approaches were used to obtain routine surveillance EMB: access via the internal jugular vein (94.7%, *n* = 7748) or via the vena femoralis (*n* = 437). The complications are summarized in Table 2. In 139/8185 (1.7%) EMB procedures, a complication occurred. Major complications were very rare (9/8185; 0.1%), including cardiac tamponade (2x), pneumothorax (6x), and hemothorax. There were no cases of severe tricuspid valve injury or death due to an EMB procedure. The remaining complications (130/8185; 1.6%) were regarded as minor complications, mainly consisting of puncture of the carotid artery in the early periods when ultrasound was not routinely used (Table 2). In 20 cases, access via the femoral vein was necessary due to a local thrombus or occlusion of the right internal jugular vein related to a surgical complication or after prolonged central cannulation. Other complications were related to failing equipment and/or logistical issues, in which the procedure was halted. Overall, the number of complications remained stable over time, and there were no differences in complication rate when comparing procedures performed early vs late after HTx (Supplementary Table 3).

**Table 2:** Procedural Complications

Complications		Complication	Frequency (% complications)	Complication rate (% of EMB procedures)
Major complications		Pneumothorax	6 (4.3)	0.07
		Tamponade	2 (1.4)	0.02
		Hemothorax	1 (0.7)	0.01
Minor complications	Vascular	Carotid artery puncture	91 (65.5)	1.11
		Failed access to the internal jugular vein	20 (14.4)	0.24
		Failed access to the femoral vein	2 (1.4)	0.02
		Femoral artery puncture	1 (0.7)	0.01
		Coronary fistula	1 (0.7)	0.01
		Air embolism	1 (0.7)	0.01
	Valvular	Chorda rupture	4 (2.9)	0.05
	Aritmogenic	RBBB	1 (0.7)	0.01
	Other	Equipment/logistics	9 (6.5)	0.11

EMB, endomyocardial biopsy; RBBB, right bundle branch block.

### Yield and harm of EMB protocols

**Figure 5:**
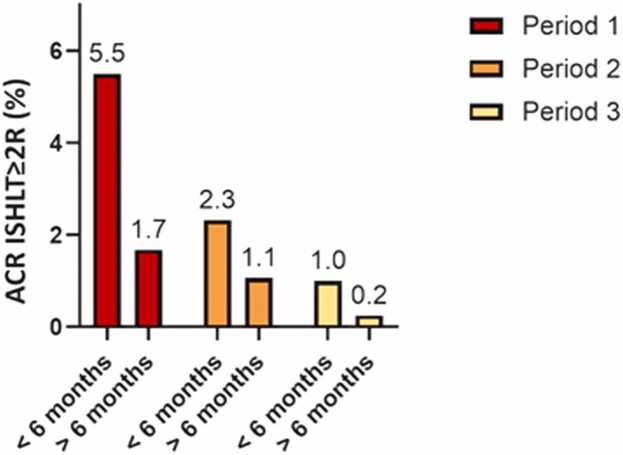
Incidence of clinically-relevant ACR (ISHLT ≥ 2R) in routine EMBs over time for early (<6 months) and late (>6 months) rejections after HTx per period. EMB, endomyocardial biopsy.

While the complication rate remained stable (Supplementary Table 3), the majority of clinically-relevant rejections occurred within the first 6 months post-HTx and decreased per period (Figure 5). The number of EMB procedures needed to detect clinically-relevant rejection (ie, number-needed-to-diagnose; NND) increased over time from 22.6 (Period 1) to 120.3 (Period 3) EMB procedures (Table 3). As the complication rate (∼2%; Supplementary Table 3) remained stable over time, the yield vs harm of EMBs became less favorable over time when looking at both the different time periods (ie, Period 1 vs Period 3) and the timing (early vs late post-HTx) after HTx.

**Table 3:** Number-Needed-to-Diagnose (NND) to Detect Clinically-Relevant Rejection (Early [<6 Months] and Late [>6 Months]) After HTx

Period		Period 1			Period 2			Period 3	
	Overall	Early	Late	Overall	Early	Late	Overall	Early	Late
NND	22.6	18.2	63.0	49.4	43.2	97.7	120.3	99.9	426.0

NND, number needed to diagnose.

## Discussion

In this retrospective single-center study, we examined the diagnostic yield and complication rate of routine surveillance EMB procedures after HTx over the past 36 years. With biopsy data from 474 patients and a total of 6363 biopsy procedures within the first year after HTx, the current analysis represents one of the largest cohorts investigating trends in ACR, including changes in frequency and duration over the last decades.

Our study has several important findings that are relevant given the changing landscape of invasive and non-invasive methods to detect rejection after HTx *(Central Illustration)*. First, the one-year ACR-free survival was 80.2% for clinically-relevant ACR (ISHLT ≥ 2R) in the total cohort of patients after HTx. Second, complications related to the EMB procedure were rare (1.7%), with the majority being classified as minor complication. Third, the incidence of clinically-relevant rejection detected in EMBs was low (2.2%), and both “any ACR” and clinically-relevant ACR decreased significantly over time, leading to an improved rejection-free survival in the most recent period. Finally, ACR mainly occurred early (within 6 months) post-HTx, while the NND increased as ACR beyond 6 months was rare.

Less-invasive methods to screen for ACR have been developed with varying success, giving rise to important questions with regard to its clinical implementation.^6^, ^16^, ^17^, ^18^, ^19^ As a result, different strategies are currently applied for the monitoring of ACR in HTx patients, and clear distinctions between the USA and Europe are apparent. Indeed, in the USA most centers have reduced the number of EMBs next to the implementation of novel detection methods, while in Europe centers continue with existing invasive EMBs protocols.^20^ Independent of the preferred method of rejection surveillance, insights on the optimal frequency and duration is necessary to safely evolve towards a less-invasive protocol.

It was reported previously that ACR episodes mainly occur in the first year after HTx.^3^, ^4^ Following this line, three periods were distinguished in the past 36 years of post-transplantation care in our center, each with a different but decreasing number of EMB procedures. Due to improved immunosuppressive regimens and encouraging outcomes, EMBs beyond the first year were reduced and later removed from our protocol after Period 2. We observed a significant improvement in rejection-free survival over time, being the highest in the most recent period. By dividing the first year after HTx into two equal parts of six months, we were able to better compare ACR episodes between the three periods. The rejection-free survival for clinically-relevant ACR after 12 months was approximately 80% for all patients and 82% by 6 months. This is in line with previous reports, showing that most rejection episodes occur within six months after HTx.^5^, ^21^

With 11.1% any ACR (ISHLT > 1R) and 2.2% clinically-relevant (ISHLT ≥ 2R) rejection in the performed, the observed frequency seems relatively low compared to previous studies reporting ISHLT ≥ 2R ranging from 4.8% to 8.5%.^22^, ^23^ A possible explanation could be related to the difference in sample size (up to 2642 EMB procedures previously). Interestingly, this difference becomes much less when looking at the early years of our heart transplant program (1980s and early 1990s) with an overlapping time period. Nevertheless, both in our cohort and in the previously mentioned studies, the majority of ACR is classified as ISHLT 1R.

Importantly, our study excluded symptom-triggered EMB procedures, and we were able to investigate trends over time in a large study population, extending previous reports. It is important to emphasize that one of the earliest but also only prospective study investigating the role of gene expression profiling vs EMB for rejection surveillance included the majority of patients (+/−70%) later than 12 months after HTx.^10^ A very recent study from Spain prospectively investigated dd-cfDNA (Allonext assay), illustrating a limited discriminatory power above the normal threshold suggesting to use non-invasive monitoring in low-risk patients only.^24^

Complication rate of EMB was relatively low (1.7%) and comparable to the recent retrospective study by Cusi et al reporting complications in 1.6% of the EMB procedures performed.^19^ Furthermore, our study further strengthens the observation that in the most recent era, chances of causing a complication are higher than detecting clinically-relevant rejection, especially after 6 months post-transplantation. The NND to detect clinically relevant rejection was 3-5 times higher after six months and increased considerably per period. This increase in NND could be due to multiple contributing factors, including advancements in immunosuppressive therapies, evolving patient characteristics, and growing expertise. However, irrespective of the reason for an increase in NND, our study shows that a low-surveillance approach may be safe.

Although it remains to be seen whether novel surveillance techniques are as sensitive as EMB, including the ability to provide prognostic and therapeutic information, the time has come to embrace the transition towards a less-invasive strategy, also given the increasing demand on health care resources in general. Our study provides data on the optimal surveillance duration and reduction in EMB procedures regarding the risk of ACR in the current era of care for heart transplant recipients. These findings support a safe transition towards less-invasive surveillance protocols and offer guidance on the timing and use of non-invasive strategies. These less-invasive protocols could still include EMBs when biomarkers are high. Taken together, routine EMB beyond 6 months may no longer be of clinical benefit in the current era of post-transplant care, which also has implications for the timing and duration of non-invasive rejection monitoring after HTx.^25^

## Limitations

Our study is limited by the retrospective single-center design. As the data was obtained from different source documents (archived health records on paper, scanned material, and electronic health record), especially the source data from the 1980s till 1990s was documented by various health care professionals, which may have introduced misinterpretation of the clinical reports. As histologic grading remained the standard for diagnosing ACR, inevitably, sampling variability and interobserver variety need to be acknowledged. Nevertheless, the decreasing number of ACR episodes over time, in line with previous studies, is reassuring with regard to the quality of the source data and with the subsequently obtained results.

## Conclusion

ACR, including clinically-relevant rejection (≥ISHLT grade 2) has declined significantly over time and is rare beyond 6 months after HTx. A conservative approach using a low-frequency screening interval seems feasible and safe, which is relevant for the transition towards a less-invasive protocol to detect ACR after HTx. Prospective studies investigating the implementation of non-invasive surveillance, especially addressing the first 6 months after HTx, are warranted to facilitate a safe transition towards a less-invasive or even EMB-free surveillance protocol.

## Declaration of Competing Interest

The authors declare that they have no known competing financial interests or personal relationships that could have appeared to influence the work reported in this paper.

## Footnotes

Appendix A Supplementary data associated with this article can be found in the online version at doi:10.1016/j.jhlto.2025.100320.
