# Supplementary material for: Clinical relevance and outcome of routine endomyocardial biopsy to detect rejection after heart transplantation
Source: JHLT Open. 2025 Jun 14;9:100320. doi: 10.1016/j.jhlto.2025.100320 (PMC12269405; doi:10.1016/j.jhlto.2025.100320)
Supplement: Supplementary file 1 — Supplementary material [file mmc1.docx]

**Supplementary material of “Clinical relevance and outcome of routine endomyocardial biopsy to detect rejection after heart transplantation”**

Authors: Leendert C. Kieviet BsC, Steven A. Muller MD, Mariusz K. Szymanski MD PhD, Manon G. van der Meer MD PhD, M. Louis Handoko MD PhD, Saskia Z.H. Rittersma MD PhD, Saskia C.A. de Jager, Egidius E. van Aarnhem MD PhD, Annelotte Vos MD, Pim van der Harst MD PhD, Linda W. van Laake MD PhD, Marish I.F.J. Oerlemans MD PHD

**Table of content**

[Table S1. Number of routine EMB procedures per Period 2](#_Toc193894785)

[Table S2. Number of routine EMB procedures and ACR incidence over time 3](#_Toc193894786)

[Table S3. Incidence of complications early (<6 months) and late (>6 months) per period 4](#_Toc193894787)

## **Table S1. Number of routine EMB procedures per Period**

| **Period** | **Time period** | **Number of routine EMB procedures** |
| --- | --- | --- |
| 1 | 1986-1994 | Sixteen in year 1 and every annual control |
| 2 | 1994-2009 | Sixteen in year 1, annually year 2-4 |
| 3 | 2009-2022 | 9-12 in year 1, no EMB >1 year |

EMB endomyocardial biopsy

## **Table S2. Number of routine EMB procedures and ACR incidence over time for early (<6 months), late (>6 months) and within the first 12 months after HTx**

| **ACR grade** | **Period 1**  **n= 1377** | | | **Period 2**  **n= 3061** | | | **Period 3**  **n= 1925** | | | **Total**  **n=6363** | | |
| --- | --- | --- | --- | --- | --- | --- | --- | --- | --- | --- | --- | --- |
| **Time** | *Early (n=999)* | *Late*  *(n=378)* | *Within 12 months*  *(n = 1377)* | *Early (n=2377)* | *Late*  *(n=684)* | *Within 12 months*  *(n = 3061)* | *Early (n=1499)* | *Late*  *(n=426)* | *Within 12 months*  *(n = 1925)* | *Early (n=4875)* | *Late*  *(n=1488)* | *Within 12 months*  *(n = 6363)* |
| **0** | 763 (76.4) | 321 (84.9) | 1084 (78.7) | 2148 (90.4) | 634 (92.7) | 2782 (90.9) | 1392 (92.9) | 400 (93.9) | 1792 (93.1) | 4303 (88.3) | 1355 (91.1) | 5658 (88.9) |
| **1R** | 181 (18.1) | 51 (13.5) | 232 (16.8) | 174 (7.3) | 43 (6.3) | 217 (7.1) | 92 (6.1) | 25 (5.9) | 117 (6.1) | 447 (9.2) | 119 (8.0) | 566 (8.9) |
| **2R** | 51 (5.1) | 6 (1.6) | 57 (4.1) | 51 (2.1) | 7 (1.0) | 58 (1.9) | 15 (1.0) | 1 (0.2) | 16 (0.8) | 117 (2.4) | 14 (0.9) | 131 (2.1) |
| **3R** | 4 (0.4) | 0 (0) | 4 (0.3) | 4 (0.2) | 0 (0) | 4 (0.1) | 0 (0) | 0 (0) | 0 (0) | 8 (0.2) | 0 (0) | 8 (0.1) |
|  |  |  |  |  |  |  |  |  |  |  |  |  |
| **≥2R** | 55 (5.5) | 6 (1.6) | 61 (4.4) | 55 (2.3) | 7 (1.0) | 62 (2.0) | 15 (1.0) | 1 (0.2) | 16 (0.8) | 125 (2.6) | 14 (0.9) | 139 (2.2) |
| **Any ACR** | 236 (23.6) | 57 (15.1) | 293 (21.2) | 229 (9.6) | 50 (7.3) | 279 (9.1) | 107 (7.1) | 26 (6.1) | 133 (6.7) | 572 (11.8) | 133 (8.9) | 705 (11.1) |

EMB endomyocardial biopsy ACR acute cellular rejection

## **Table S3. Incidence of complications early (<6 months) and late (>6 months) per period. (<1 year after HTx)**

| Complication (%) | Period 1 | | Period 2 | | Period 3 | |
| --- | --- | --- | --- | --- | --- | --- |
|  | early | late | early | late | early | late |
| Yes | 12 (1.2) | 6 (1.6) | 46 (1.9) | 15 (2.2) | 29 (1.9) | 8 (1.9) |
| No | 993 (98.8) | 371 (98.4) | 2354 (98.1) | 673 (97.8) | 1489 (98.1) | 422 (98.1) |

HTx heart transplantation
